# 2022 American Society of Metabolic and Bariatric Surgery (ASMBS) and International Federation for the Surgery of Obesity and Metabolic Disorders (IFSO) Indications for Metabolic and Bariatric Surgery

**DOI:** 10.1007/s11695-022-06332-1

**Published:** 2022-11-07

**Authors:** Dan Eisenberg, Scott A. Shikora, Edo Aarts, Ali Aminian, Luigi Angrisani, Ricardo V. Cohen, Maurizio de Luca, Silvia L. Faria, Kasey P.S. Goodpaster, Ashraf Haddad, Jacques M. Himpens, Lilian Kow, Marina Kurian, Ken Loi, Kamal Mahawar, Abdelrahman Nimeri, Mary O’Kane, Pavlos K. Papasavas, Jaime Ponce, Janey S. A. Pratt, Ann M. Rogers, Kimberley E. Steele, Michel Suter, Shanu N. Kothari

**Affiliations:** 1 Department of Surgery, Stanford School of Medicine, VA Palo Alto Health Care System, 3801 Miranda Avenue, GS 112, Palo Alto, CA 94304 USA; 2 Department of Surgery, Center for Metabolic and Bariatric Surgery, Brigham and Women’s Hospital, and Harvard Medical School, Boston, MA USA; 3 WeightWorks Clinics and Allurion Clinics, Amersfoort, The Netherlands; 4 Bariatric and Metabolic Institute, Cleveland Clinic, Cleveland, OH USA; 5 Department of Public Health, University of Naples Federico II, Naples, Italy; 6 Center for the Treatment of Obesity and Diabetes, Hospital Alemão Oswaldo Cruz, Sao Paolo, Brazil; 7 Department of Surgery, Rovigo Hospital, Rovigo, Italy; 8 Gastrocirurgia de Brasilia, University of Brasilia, Brasilia, Brazil; 9 Gastrointestinal Bariatric and Metabolic Center, Jordan Hospital, Amman, Jordan; 10 Department of Surgery, Delta CHIREC Hospital, Brussels, Belgium; 11 Adelaide Bariatric Centre, Flinders University of South Australia, Adelaide, Australia; 12 Department of Surgery, New York University Grossman School of Medicine, New York, NY USA; 13 St. George Hospital and Sutherland Hospital, Kogarah, New South Wales Australia; 14 Department of General Surgery, Sunderland Royal Hospital, Sunderland, UK; 15 Department of Surgery, Carolinas Medical Center, University of North Carolina, Charlotte, NC USA; 16 Department of Nutrition and Dietetics, Leeds Teaching Hospitals NHS Trust, Leeds, UK; 17 Division of Metabolic and Bariatric Surgery, Hartford Hospital, Hartford, CT USA; 18 Bariatric Surgery Program, CHI Memorial Hospital, Chattanooga, TN USA; 19 Division of Pediatric Surgery, Lucille Packard Children’s Hospital, Palo Alto, CA USA; 20 Department of Surgery, Penn State Health Milton S. Hershey Medical Center, Hershey, PA USA; 21 NIDDK Metabolic and Obesity Research Unit, National Institutes of Health, Bethesda, MD USA; 22 Department of Surgery, Riviera-Chablais Hospital, Rennaz, Switzerland; 23 Department of Visceral Surgery, University Hospital, Lausanne, Switzerland; 24 Prisma Health, Department of Surgery, University of South Carolina School of Medicine, Greenville, SC USA

**Keywords:** Obesity, Metabolic and bariatric surgery, IFSO, ASMBS, Criteria, Indications

## Abstract

Metabolic and bariatric surgery (MBS) is recommended for individuals with a body mass index (BMI) >35 kg/m^2^, regardless of presence, absence, or severity of co-morbidities.

MBS should be considered for individuals with metabolic disease and BMI of 30-34.9 kg/m^2^.

BMI thresholds should be adjusted in the Asian population such that a BMI >25 kg/m^2^ suggests clinical obesity, and individuals with BMI >27.5 kg/m^2^ should be offered MBS.

Long-term results of MBS consistently demonstrate safety and efficacy.

Appropriately selected children and adolescents should be considered for MBS.

(Surg Obes Relat Dis 2022; 10.1016/j.soard.2022.08.013) © 2022 American Society for Metabolic and Bariatric Surgery. All rights reserved.

Thirty years ago, the National Institutes of Health (NIH) convened a Consensus Development Conference that published a Statement on gastrointestinal surgery for severe obesity, reflecting expert assessment of the medical knowledge available at the time [1]. Specifically, it sought to address “the surgical treatments for severe obesity and the criteria for selection, the efficacy and risks of surgical treatments for severe obesity, and the need for future research on and epidemiological evaluation of these therapies,” and included specific recommendations for practice. Among these are that nonsurgical programs should be initial therapy for severe obesity; that patients should be carefully selected for surgery after evaluation by a multidisciplinary team; and that lifelong medical surveillance continue after surgery. The 1991 NIH Consensus Statement has been used by providers, hospitals, and insurers, as a standard for selection criteria for bariatric surgery. A body mass index (BMI) >40 kg/m^2^, or BMI >35 kg/m^2^ with co-morbidities, is a threshold for surgery that is applied universally.

Since its publication, hundreds of studies have been published on the worldwide obesity epidemic and global experience with metabolic and bariatric surgery (MBS), which has greatly enhanced the understanding of obesity and its treatment [2, 3]. Now recognized as a chronic disease, obesity is associated with a chronic low-grade inflammatory state and immune dysfunction [4, 5]. It is suspected that the prolonged state of inflammation leads to a disruption of homeostatic mechanisms and consequently to metabolic disorders commonly associated with obesity, mediated by incompletely elucidated pathways involving cytokine production, adipokines, hormones, and acute-phase reactants [5–8].

With an increasing global MBS experience, long-term studies have proven it an effective and durable treatment of severe obesity and its co-morbidities. Studies with long-term follow up, published in the decades following the 1991 NIH Consensus Statement, have consistently demonstrated that MBS produces superior weight loss outcomes compared with nonoperative treatments [9–14]. After surgery, the significant improvement of metabolic disease, as well as the decrease in overall mortality, has been reported in multiple studies further supporting the importance of this treatment modality [15–19]. Concurrently, the safety of bariatric surgery has been studied and reported extensively [20–23]. Perioperative mortality is very low, ranging between .03% and .2% [24]. Thus, it is not surprising that MBS has become one of the most commonly performed operations in general surgery [25].

The operations commonly performed have evolved as well. Older surgical operations have been replaced with safer and more effective operations. The 1991 NIH Consensus Statement described the vertical banded gastroplasty (VBG) and Roux-en-Y gastric bypass (RYGB) as the dominant procedures in clinical practice at the time. Currently, the dominant procedures are sleeve gastrectomy and RYGB, together accounting for approximately 90% of all operations performed worldwide [26], and each has well-studied mid- and long-term outcomes. Other operations performed include adjustable gastric banding (AGB), biliopancreatic diversion with duodenal switch, and one-anastomosis gastric bypass. The VBG is of historical interest and no longer performed, and the popularity of the AGB has diminished significantly over the past decade. MBS is now preferably performed using minimally invasive surgical approaches (laparoscopic or robotic assisted).

In light of significant advances in the understanding of the disease of obesity, its management in general, and metabolic and bariatric surgery specifically, the leaderships of the American Society of Metabolic and Bariatric Surgery (ASMBS) and the International Federation for the Surgery of Obesity and Metabolic Disorders (IFSO) have convened to produce this joint statement on the current available scientific information on metabolic and bariatric surgery and its indications.

## Criteria for surgery

### ***BMI***

Despite limitations of BMI to accurately risk stratify patients with obesity for their future health risk, it is the most feasible and widely used criteria to identify and classify patients with overweight or obesity. MBS is currently the most effective evidence-based treatment for obesity across all BMI classes.

BMI 30–34.9 kg/m^2^. Class I obesity (BMI 30–34.9 kg/m^2^) is a well-defined disease that causes or exacerbates multiple medical and psychological co-morbidities, decreases longevity, and impairs quality of life. Prospective and large retrospective studies support the notion that MBS should be considered a treatment option for patients with class I obesity who do not achieve substantial or durable weight loss or co-morbidity improvement with nonsurgical methods, and early findings prompted international diabetes organizations to publish a joint statement supporting the consideration of MBS for patients with BMI <35 kg/m^2^ and type 2 diabetes (T2D) [27]. Aminian et al. [28] summarize the available data from randomized controlled trials (RCT’s), meta-analyses, and observational studies that also include individuals with BMI <35 kg/m^2^. These data consistently demonstrate the weight loss and metabolic benefits of MBS in individuals with class I obesity [28]. Noun et al. [29] reported on >500 consecutive patients with BMI <35 kg/m^2^ who had MBS and demonstrated significant weight loss at 5 years and improvement or remission of diabetes, hypertension, and dyslipidemia. In a cohort study of more than 1000 patients, MBS in individuals with BMI <35 kg/m^2^ produced high rates of co-morbidity remission and was more likely than MBS in BMI >35 kg/m^2^ to achieve BMI <25 kg/m^2^ [30]. Ikramuddin et al. [31] and Schauer et al. [32] demonstrated superior diabetes improvement and weight loss following MBS in randomized controlled trials that include the subset of patients with BMI <35 kg/m^2^. A 3-arm randomized controlled trial that had 43% of its subjects with class I obesity, demonstrated that MBS is superior to lifestyle intervention for remission of T2D, 3 years after surgery [33].

Furthermore, randomized trials designed specifically to study the population with BMI <35 kg/m^2^ also demonstrate significant benefits of MBS in individuals with class I obesity compared with other treatment. O’Brien et al. [34], in a randomized controlled trial of 80 patients with BMI 30–35 kg/m^2^ assigned to nonsurgical treatment or MBS, demonstrated that patients undergoing MBS had superior long-term weight reduction and improvement of metabolic disease. A short-term follow-up randomized trial examining patients with T2D demonstrated significantly improved remission of diabetes and weight loss in those individuals undergoing MBS compared with medical weight management [35]. In a study of 51 patients with class I obesity diabetes randomized to either medical therapy or medical therapy plus MBS, the cohort who underwent surgery has superior diabetes control up to 2 years postoperatively [36].

Medical weight loss is considered to have greater durability in individuals with BMI <35 kg/m^2^ than individuals with BMI >35 kg/m^2^, and thus it is recommended that a trial of nonsurgical therapy is attempted before considering surgical treatment. However, if attempts at treating obesity and obesity-related co-morbidities such as T2D, hypertension, dyslipidemia, obstructive sleep apnea, cardiovascular disease (e.g., coronary artery disease, heart failure, atrial fibrillation), asthma, fatty liver disease and nonalcoholic steatohepatitis, chronic kidney disease, polycystic ovarian syndrome, infertility, gastroesophageal reflux disease, pseudotumor cerebri, and bone and joint diseases have not been effective, MBS should be considered for suitable individuals with class I obesity [27, 28, 37, 38].

BMI >35 kg/m^2^. Given the presence of high-quality scientific data on safety, efficacy, and cost-effectiveness of MBS in improving survival and quality of life in patients with BMI ≥35 kg/m^2^, MBS should be strongly recommended in these patients regardless of presence or absence of evident obesity-related co-morbidities. Current nonsurgical treatment options for patients with BMI ≥35 kg/m^2^ are ineffective in achieving a substantial and sustained weight reduction necessary to significantly improve their general health. Physical problems related to excess body weight, undiagnosed obesity-related co-morbidities, risk of developing obesity-related co-morbidities in the future, and impaired quality of life related to physical and mental consequences of obesity threaten the general health of individuals with moderate to severe obesity even in the absence of diagnosed obesity-related co-morbidities [27, 28]. Thus, MBS is recommended in this population.

### ***BMI thresholds in the Asian population***

The World Health Organization defines the terms *overweight* and *obesity* based on BMI thresholds [39]. In its consensus panel statement of 1991, the NIH stated that the “risk for morbidity linked with obesity is proportional to the degree of overweight.” However, BMI does not account for an individual’s sex, age, ethnicity, or fat distribution, and is recognized as only an approximation of adiposity. The health risk in a patient with BMI 30 kg/m^2^ with visceral and ectopic fat accumulation and subsequent metabolic and cardiovascular disease would be significantly higher than a patient with BMI 40 kg/m^2^ whose adipose tissue is mainly accumulated in the lower extremity. In the Asian population the prevalence of diabetes and cardiovascular disease is higher at a lower BMI than in the non-Asian population. Thus, BMI risk zones should be adjusted to define obesity at a BMI threshold of 25–27.5 kg/m^2^ in this population. Therefore, in certain populations access to MBS should not be denied solely based on traditional BMI thresholds [28, 37, 40–44].

## Extremes of age

### ***Older population***

Coincident with the demonstrated safety of MBS, surgery has been performed successfully in increasingly older patients over the past few decades, including individuals >70 years of age [45, 46]. In septuagenarians MBS is associated with slightly higher rates of postoperative complications compared with a younger population, but still provides substantial benefits of weight loss and remission of co-morbid disease [46]. In fact, the presence of obesity co-morbid disease and the choice of operation are more predictive of 30-day adverse outcomes than age alone [47]. Similar to other operations, the question of whether there should be an upper chronologic age limit is complex. The physiologic changes that occur with aging may have an impact on the efficacy of MBS, the incidence of postoperative complications, and the ability of older patients to recover from surgery. However, it appears that factors other than age, such as frailty, cognitive capacity, smoking status, and end-organ function have an important role [48].

Frailty, rather than age alone, is independently associated with higher rates of postoperative complications following MBS [49]. Furthermore, when considering MBS in older patients, the risk of surgery should be evaluated against the morbidity risk of obesity-related diseases. Thus, there is no evidence to support an age limit on patients seeking MBS, but careful selection that includes assessment of frailty is recommended.

### ***Pediatrics and adolescents***

Children and adolescents with obesity carry the burden of the disease and its co-morbidities into adulthood, increasing the individual risk for premature mortality and complications from obesity co-morbidities [50].

MBS is safe in the population younger than 18 years and produces durable weight loss and improvement in co-morbid conditions. Adolescents with severe obesity undergoing RYGB have significantly greater weight loss and improvement of cardiovascular co-morbidities compared with adolescents undergoing medical management [51]. Furthermore, improvement in hypertension and dyslipidemia has been demonstrated up to 8 years after surgery [52]. Additional studies from the prospective Teen-Longitudinal Assessment of Bariatric Surgery database (Teen-LABS) demonstrated significant weight loss and durable improvement in cardiovascular risk factors and T2D in adolescents undergoing MBS. Furthermore, data suggest that the benefits of RYGB on T2D and hypertension are greater in adolescents than adults [52–55]. Prospective data shows durable weight loss and maintained co-morbidity remission in patients as young as 5 years old [56].

The American Academy of Pediatrics and the ASMBS recommend consideration of MBS in children/adolescents with BMI >120% of the 95th percentile (class II obesity) and major co-morbidity, or a BMI >140% of the 95th percentile (class III obesity) [57, 58]. In addition, MBS does not negatively impact pubertal development or linear growth, and therefore a specific Tanner stage and bone age should not be considered a requirement for surgery [56]. Increasingly, syndromic obesity, developmental delay, autism spectrum, or history of trauma is not considered a contraindication to MBS in adolescents [59].

## Bridge to other treatment

### ***Joint arthroplasty***

Poorer outcomes after total joint arthroplasty have been associated with obesity, such that some orthopedic surgical societies discourage hip and knee replacement in individuals with BMI >40 kg/m^2^ [60–62]. In addition to the technical challenge of performing orthopedic surgery in individuals with severe obesity, patients with obesity undergoing joint arthroplasty are at increased risk of hospital readmission and surgical complications, such as wound infection and deep vein thrombosis [63–67].

There are reports to suggest that MBS may be effective as a bridge to total joint arthroplasty in individuals with class II/III obesity when performed >2 years prior to joint surgery [68, 69]. A study of veterans with osteoarthritis demonstrated that an average of 35 months elapsed between MBS and joint arthroplasty or lumbar spine surgery in patients with known osteoarthritis [70]. MBS prior to total knee and hip arthroplasty has been shown to decrease operative time, hospital length-of-stay, and early postoperative complications [66, 71, 72]. Long-term joint-related complications rates were not significantly different.

In a randomized clinical trial on 82 patients with obesity and osteoarthritis, 41 were randomized to AGB 12-months prior to total knee arthroplasty (TKA) and 41 were randomized to receive usual nonoperative weight management prior to TKA. In a median follow-up of 2 years after TKA, 14.6% of patients in the MBS group incurred the primary outcome of composite complications, compared with 36.6% in the control (non-MBS) group (difference 22.0%, *P* = .02). Interestingly, TKA was declined by 29.3% of subjects in the MBS group because of symptom improvement following weight loss, compared with only 4.9% in the control group [73].

### ***Abdominal wall hernia repair***

Obesity is a risk factor for the development of ventral hernia. It increases the risk for impaired wound healing, local and systemic infections, and other complications following hernia repair, and increases the risk for recurrence [74–76]. In addition to a larger volume of subcutaneous soft tissue, abdominal wall hernias in the population with obesity tend to be larger, adding to the complexity of repair in these patients. While the timing of MBS relative to hernia repair remains controversial, evidence suggests that patients with large, chronic abdominal wall hernia may benefit from significant weight loss initially as staged procedure to definitive hernia repair [75, 77]. Thus, in patients with severe obesity and an abdominal wall hernia requiring elective repair, MBS should be considered first to induce significant weight loss, and consequently reduce the rate of complications associated with hernia repair and increase durability of the repair.

### ***Organ transplantation***

Class III obesity is associated with end-stage organ disease and may limit the access to transplantation of the patient with obesity, since it is a relative contraindication for solid organ transplantation and poses specific technical challenges during surgery. Conversely, MBS may be overlooked as an option in patients with severe end-stage organ disease. Nonetheless, MBS has been described in patients with end-stage organ disease as a way to improve their candidacy for transplantation. Patients with end-stage organ disease can achieve meaningful weight loss and improve their eligibility to receive an organ transplant [78]. Studies suggest that more than 50% of patients with end-stage renal disease (ESRD) and morbid obesity are able to be listed for kidney transplant within 5 years after MBS [79]. Similarly, MBS is shown to be safe and effective as a bridge to liver transplantation in selected patients who would otherwise be ineligible [80, 81]. Heart transplant candidacy can also be improved by MBS, and reports in some patients demonstrate significant improvement in left ventricular ejection fraction after surgery to remove the requirement for transplantation [82, 83]. MBS has been shown to be safe and effective in patients with heart failure and a left ventricular assist device (LVAD). McElderry et al. [84] demonstrated in a study of 2798 patients who underwent LVAD implantation that a history of prior MBS was associated with a 3-fold higher probability of heart transplantation in follow-up, compared with patients who did not have MBS. In addition, limited data suggest that patients with obesity and end-stage lung disease may lose sufficient weight after MBS to achieve listing for transplantation [85].

## MBS in the high-risk patient

### ***BMI > 60 kg/m***^***2***^

There is no consensus concerning the best procedure for individuals with especially high BMI, but the efficacy and safety of MBS have been demonstrated in this population [86, 87]. In general, mortality risk increases with increasing BMI, and BMI >50 kg/m^2^ has been implicated in increasing surgical risk in older studies [88–90]. Individuals with BMI >60 kg/m^2^ are considered to be at especially high risk for surgery since these patients have greater obesity-associated disease burden and more challenging surgical anatomy, resulting in longer operative times, higher rates of perioperative morbidity, and longer hospital lengths of stay in some studies [91, 92]. Others, however, failed to demonstrate a significant difference in perioperative complications, length of stay, 30-day mortality, or long-term outcomes after MBS when individuals with BMI >60 kg/m^2^ were compared with those with BMI <60 kg/m^2^. Furthermore, studies have shown that MBS can be performed safely in patients with BMI >70 kg/m^2^ [93]. Therefore, MBS should be considered as a preferred method to achieve clinically significant weight loss in patients with extreme BMI.

### ***Cirrhosis***

Obesity is a significant risk factor for nonalcoholic fatty liver disease (NAFLD), nonalcoholic steatohepatitis (NASH), and consequent cirrhosis. At the same time, obesity conveys a 3-fold increase in the risk of liver decompensation in patients with known cirrhosis [94]. In addition to inducing significant and durable weight loss, MBS has been association with histologic improvement of NASH and regression of fibrosis in early cases, leading to a reduced risk of hepatocellular carcinoma [94, 95]. Furthermore, MBS is associated with an 88% risk reduction of progression of NASH to cirrhosis [18].

The patient with obesity and compensated cirrhosis is at higher risk for perioperative mortality following MBS, but the risk remains small (<1%) and the benefits significant [94, 96, 97]. There is a paucity of data on surgical outcomes in patients with clinically significant portal hypertension [98]. Careful patient selection and consideration of choice of surgical procedure are important to ensure best outcomes.

### ***Heart failure***

There are increasing data to suggest that MBS can be a useful adjunct to treatment in patients with obesity and heart failure before heart transplantation or placement of a left ventricular assist device (LVAD), and performed with low morbidity and mortality [82, 84, 99]. The consequent improvement in obesity and associated co-morbidities improves overall health and can reduce the future risk associated with cardiac therapies. Furthermore, limited studies have shown that MBS in individuals with heart failure was associated with a significant improvement of left ventricular ejection fraction (LVEF), improvement of functional capacity, and higher chances for receiving heart transplantation [84, 100–102].

## ***Patient evaluation***

The 1991 NIH Consensus Statement recommends that patients who are candidates for MBS should be evaluated by a “multidisciplinary team with access to medical, surgical, psychiatric, and nutritional expertise” [1]. The value of assessments by such a team has since been reiterated [103–105], reflecting the recognition of the complexity of the disease of obesity, and the ability to provide a comprehensive risk/benefit analysis when considering MBS. This may also facilitate the patient’s ability to comprehend the life-long changes that can be expected after surgery, benefitting from the expertise of different healthcare providers [106]. Studies have suggested that the addition of a multidisciplinary team to the perioperative care of the patient may decrease rates of complications [107, 108].

While there has been initial enthusiasm for weight loss prior to surgery, there are no data to support the practice of insurance-mandated preoperative weight loss; this practice is understood to be discriminatory, arbitrary, and scientifically unfounded, contributing to patient attrition, unnecessary delay of lifesaving treatment, and progression of life-threatening co-morbid conditions [109]. A multidisciplinary team can help assess and manage the patient’s modifiable risk factors with a goal of reducing risk of perioperative complications and improving outcomes; the decision for surgical readiness should be primarily determined by the surgeon.

The nutritional status of patients seeking MBS is important [104, 110]. A nutritional assessment by a registered dietitian with expertise in MBS can help obtain a comprehensive weight history, identify maladaptive eating behaviors or patterns, and correct any micronutrient deficiencies prior to surgery. A registered dietitian can also provide preoperative nutrition education and prepare the patient for expected dietary changes after MBS [103, 104]. In addition, a registered dietitian with expertise in MBS can assist in the management of postoperative patients who may be experiencing food intolerances, malabsorption issues and micronutrient deficiencies, and weight regain.

Mental health conditions such as depression and binge eating disorders, as well as substance abuse, are found at higher rates among candidates for MBS than in the general population. The pre-surgical evaluation process is designed to optimize surgical outcomes and implement interventions that can address disordered eating, severe uncontrolled mental illness, or active substance abuse. Licensed mental health providers with specialty knowledge and experience in MBS behavioral health are important to assess patients for psychopathology, and determine the candidate’s ability to cope with the adversity of surgery, changing body image, and life-style changes required after MBS. In addition, stressors that may affect long-term outcomes such as financial, housing and food insecurity should be identified [104, 111].

## Outcomes

### ***Weight loss and co-morbidity improvement***

The ASMBS established standard guidelines for reporting on outcomes of MBS, including weight loss, co-morbidity remission, surgical complications, and quality of life [112]. Mid- and long-term outcomes of MBS, confirming the safety, efficacy and durability of surgery are extensively studied and reported in the literature [24, 113].

Overall weight loss outcomes for MBS that are durable for years after surgery are consistently reported at greater than 60% percent excess weight loss (%EWL), with some variation depending on the specific operation performed [14, 114, 115]. MBS is proven superior to diet, exercise, and other lifestyle interventions in attaining significant and durable weight loss, and improving obesity-related co-morbid conditions in multiple observational and prospective studies [9, 32, 116]. Durability of weight loss at 5, 10, and 20 years after surgery has been consistently demonstrated in multiple studies [10, 11, 14, 32, 117].

Obesity is associated with diseases affecting nearly every organ system. They include the cardiovascular system (hypertension, dyslipidemia, coronary artery disease, heart failure, stroke), respiratory system (obstructive sleep apnea, asthma), digestive system (gastroesophageal reflux disease, gallbladder disease, pancreatitis), endocrine system (insulin resistance, T2D), reproductive system (polycystic ovary syndrome, infertility), liver (NAFLD, NASH), kidneys (nephrolithiasis, chronic kidney disease), musculoskeletal system (osteoarthritis) and mental health [118]. Nearly all of these conditions have demonstrated improvement, and in some cases remission, after weight loss associated with MBS. There is substantial evidence demonstrating the significant and durable clinical improvement of metabolic syndrome following surgery. In a large cohort study of >180,000 Medicare beneficiaries, patients who underwent MBS had significantly lower risk of new-onset heart failure, myocardial infarction, and stroke, compared with matched controls at 4 years after surgery [119]. The long-term reduction in cardiovascular risk after MBS has been shown by others, especially in individuals with concurrent T2D [19, 120].

Greater weight loss and improvement in T2D, hypertension, and dyslipidemia has been demonstrated beyond 10 years after MBS, compared with nonsurgical controls [10, 121]. Sustained weight loss of at least 15% is recognized as having a significant effect on inducing marked improvement of metabolic derangement in most patients, with individuals undergoing MBS demonstrating a consistent and durable benefit [122]. In the randomized controlled STAMPEDE trial, medical therapy with RYGB or sleeve gastrectomy were shown to be superior to medical therapy alone in the long-term treatment of T2D [32]. Similarly, Mingrone et al. [123] demonstrated in a randomized controlled trial the superiority of MBS to medical therapy in the management of type 2 diabetes 5 years after surgery. Others have shown that microvascular complications of diabetes are decreased after MBS with up to 20 years follow up [116], and that the risk for, and markers of diabetic nephropathy improve after MBS in retrospective and randomized prospective studies [124–127].

### ***Cancer risk***

Obesity is associated with an elevated risk of multiple cancers, including esophagus, breast, colorectal, endometrial, gallbladder, stomach, kidney, ovary, pancreas, liver, thyroid, multiple myeloma, and meningioma [128–133]. There is evidence to suggest that MBS can lead to a significant reduction in incidence of obesity-associated cancer and cancer-related mortality, compared with obese individuals who did not undergo surgery. Multiple studies have shown that MBS reduces the risk of developing cancer in the population with class II/III obesity, ranging from 11% to 50% for all cancer types [130, 134–137]. Benefits were also documented for the incidence of specific cancers, such as gastrointestinal and hepatobiliary cancers, genitourinary cancers, and gynecological cancers.

Furthermore, MBS may significantly reduce overall cancer mortality compared with nonsurgical obese controls [134, 137]. There is some evidence to suggest that the risk-reduction attenuates as time from surgery increases, although it is unclear to what extent type of operation, type of cancer, health behaviors, and presence of co-morbidities confound these findings [138]. Nonetheless, a recent retrospective cohort study of >30,000 patients with a median follow-up of 6 years found that adults with obesity who underwent MBS had a 32% lower risk of developing cancer and 48% lower risk of cancer-related death compared with a matched cohort who did not have surgery [137].

### ***Mortality***

Large prospective and retrospective studies have consistently reported the lower mortality and improved survival benefit of MBS. Representative studies include the Swedish Obese Subjects study demonstrated an adjusted decreased overall mortality by 30.7% in the group of 2010 surgical patients compared with nonsurgical controls, at an average of 10 years after surgery [17]. Similar results were demonstrated in a large retrospective study comparing 9949 individuals who had undergone RYGB compared with nonsurgical controls [139]. With a mean follow-up of 7 years, adjusted overall mortality decreased by 40% in the MBS group. In a retrospective cohort study of 2500 mostly male patients, all-cause mortality was significantly lower at 5-10 years after MBS compared to controls [16]. In a large meta-analysis with an overall >170,000 subjects, median life-expectancy was increased by 6.1 years after MBS compared with usual care [140]. In this study, the median life-expectancy is increased further in the population with diabetes. A study of Medicare beneficiaries comparing >94,000 individuals who had MBS to matched controls demonstrated a significantly lower risk of mortality [119]. Thus, the durable benefits of MBS for individuals with class II/III obesity are reflected in an overall lower mortality years after surgery in multiple populations.

### ***Revisional surgery***

With the rise in the number of metabolic and bariatric operations performed worldwide, and with the recognition of obesity as a chronic, relapsing, multifactorial disease, comes a rise in the need for revisional surgery. Indications for revisional MBS vary among individual patients, but may include weight regain, insufficient weight loss, insufficient improvement of co-morbidities, and managing complications (e.g., gastroesophageal reflux) [141–144].

Surgical revision can take the form of converting from one kind of MBS operation to another, enhancing the effect of a specific operation (e.g., distalization after RYGB), treating possible complications of the index operation, or restoring normal anatomy if possible [144, 145]. Furthermore, with the understanding of severe obesity to be a chronic disease there has been a growing recognition of the requirement for long-term management of excess weight and obesity co-morbidities. This often takes the form of multimodal therapy that could include additional or “revisional” surgery, to achieve optimal outcomes. Thus, revisional surgery may also serve as escalation therapy for those individuals who are deemed poor responders to the initial operation.

The complexity of revisional surgery is higher than primary MBS, and is associated with increased hospital length of stay, and higher rates of complications [146]. Nonetheless, revisional MBS is effective in achieving additional weight loss and co-morbidity reduction after the primary operation in selected patients, with acceptable complication rates, and low mortality rates [145, 147, 148].

## Conclusion

- Since the NIH published its statement on gastrointestinal surgery for severe obesity in 1991, the understanding of obesity and MBS has significantly grown based on a large body of clinical experience and research.
- Long-term data consistently demonstrate the safety, efficacy, and durability of MBS in the treatment of clinically severe obesity and its co-morbidities, with a resultant decreased mortality compared with nonoperative treatment methods.
- MBS is recommended for individuals with BMI >35 kg/m^2^, regardless of presence, absence, or severity of co-morbidities.
- MBS is recommended in patients with T2D and BMI >30 kg/m^2^.
- MBS should be considered in individuals with BMI of 30–34.9 kg/m^2^ who do not achieve substantial or durable weight loss or co-morbidity improvement using nonsurgical methods.
- Obesity definitions using BMI thresholds do not apply similarly to all populations. Clinical obesity in the Asian population is recognized in individuals with BMI >25 kg/m^2^. Access to MBS should not be denied solely based on traditional BMI risk zones.
- There is no upper patient-age limit to MBS. Older individuals who could benefit from MBS should be considered for surgery after careful assessment of co-morbidities and frailty.
- Carefully selected individuals considered higher risk for general surgery may benefit from MBS.
- Children and adolescents with BMI >120% of the 95th percentile and a major co-morbidity, or a BMI >140% of the 95th percentile, should be considered for MBS after evaluation by a multidisciplinary team in a specialty center.
- MBS is an effective treatment of clinically severe obesity in patients who need other specialty surgery, such as joint arthroplasty, abdominal wall hernia repair, or organ transplantation.
- Consultation with a multidisciplinary team can help manage the patient’s modifiable risk factors with a goal of reducing risk of perioperative complications and improving outcomes. The ultimate decision for surgical readiness should be determined by the surgeon.
- Severe obesity is a chronic disease requiring long-term management after primary MBS. This may include revisional surgery or other adjuvant therapy to achieve desired treatment effect.
