# Publisher Correction: 2022 American Society of Metabolic and Bariatric Surgery (ASMBS) and International Federation for the Surgery of Obesity and Metabolic Disorders (IFSO) Indications for Metabolic and Bariatric Surgery

**DOI:** 10.1007/s11695-022-06369-2

**Published:** 2022-11-29

**Authors:** Dan Eisenberg, Scott A. Shikora, Edo Aarts, Ali Aminian, Luigi Angrisani, Ricardo V. Cohen, Maurizio de Luca, Silvia L. Faria, Kasey P. S. Goodpaster, Ashraf Haddad, Jacques M. Himpens, Lilian Kow, Marina Kurian, Ken Loi, Kamal Mahawar, Abdelrahman Nimeri, Mary O’Kane, Pavlos K. Papasavas, Jaime Ponce, Janey S. A. Pratt, Ann M. Rogers, Kimberley E. Steele, Michel Suter, Shanu N. Kothari

**Affiliations:** 1 Department of Surgery, Stanford School of Medicine, VA Palo Alto Health Care System, 3801 Miranda Avenue, GS 112, Palo Alto, CA 94304 USA; 2 Department of Surgery, Center for Metabolic and Bariatric Surgery, Brigham and Women’s Hospital, and Harvard Medical School, Boston, MA USA; 3 WeightWorks Clinics and Allurion Clinics, Amersfoort, The Netherlands; 4 Bariatric and Metabolic Institute, Cleveland Clinic, Cleveland, OH USA; 5 Department of Public Health, University of Naples Federico II, Naples, Italy; 6 Center for the Treatment of Obesity and Diabetes, Hospital Alemão Oswaldo Cruz, Sao Paolo, Brazil; 7 Department of Surgery, Rovigo Hospital, Rovigo, Italy; 8 Gastrocirurgia de Brasilia, University of Brasilia, Brasilia, Brazil; 9 Gastrointestinal Bariatric and Metabolic Center, Jordan Hospital, Amman, Jordan; 10 Department of Surgery, Delta CHIREC Hospital, Brussels, Belgium; 11 Adelaide Bariatric Centre, Flinders University of South Australia, Adelaide, Australia; 12 Department of Surgery, New York University Grossman School of Medicine, New York, NY USA; 13 St. George Hospital and Sutherland Hospital, Kogarah, NSW Australia; 14 Department of General Surgery, Sunderland Royal Hospital, Sunderland, UK; 15 Department of Surgery, Carolinas Medical Center, University of North Carolina, Charlotte, NC USA; 16 Department of Nutrition and Dietetics, Leeds Teaching Hospitals NHS Trust, Leeds, UK; 17 Division of Metabolic and Bariatric Surgery, Hartford Hospital, Hartford, CT USA; 18 Bariatric Surgery Program, CHI Memorial Hospital, Chattanooga, TN USA; 19 Division of Pediatric Surgery, Lucille Packard Children’s Hospital, Palo Alto, CA USA; 20 Department of Surgery, Penn State Health Milton S. Hershey Medical Center, Hershey, PA USA; 21 NIDDK Metabolic and Obesity Research Unit, National Institutes of Health, Bethesda, MD USA; 22 Department of Surgery, Riviera-Chablais Hospital, Rennaz, Switzerland; 23 Department of Visceral Surgery, University Hospital, Lausanne, Switzerland; 24 Department of Surgery, Prisma Health, University of South Carolina School of Medicine, Greenville, SC USA

**Publisher Correction: Obesity Surgery**

**https://doi.org/10.1007/s11695-022-06332-1**

The article 2022 American Society of Metabolic and Bariatric Surgery (ASMBS) and International Federation for the Surgery of Obesity and Metabolic Disorders (IFSO) Indications for Metabolic and Bariatric Surgery, written by Dan Eisenberg et al., was originally published electronically on the publisher’s internet portal on November 6, 2022, without open access. With the author(s)’ decision to opt for Open Choice, the copyright of the article changed on November 23, 2022, to © The author(s). Published by Elsevier Inc on behalf of American Society for Metabolic & Bariatric Surgery (ASMBS) and Springer Nature on behalf of International Federation for the Surgery of Obesity and Metabolic Disorders (IFSO) 2022 and the article is forthwith distributed under a Creative Commons Attribution CC BY license.

